# Effectiveness of the Elos 2.0 prevention programme for the reduction of problem behaviours and promotion of social skills in schoolchildren: study protocol for a cluster-randomized controlled trial

**DOI:** 10.1186/s13063-021-05408-0

**Published:** 2021-07-20

**Authors:** Marília Mariano, Anderson Ribeiro da Silva, Jacqueline L. S. Lima, Nícolas Tenedine de Pinho, Hugo Cogo-Moreira, Márcia H. S. Melo, Jair J. Mari, Zila M. Sanchez, Sheila C. Caetano

**Affiliations:** 1 Department of Psychiatry, Universidade Federal de São Paulo, São Paulo, Brazil; 2 School of Public Health, The University of Hong Kong, Hong Kong, Hong Kong SAR, China; 3 Department of Clinical Psychology, University of São Paulo, São Paulo, Brazil; 4 Department of Preventive Medicine, Universidade Federal de São Paulo, São Paulo, Brazil

**Keywords:** School-based intervention, Mental health, School interactions, Child, Structured running programme

## Abstract

**Background:**

Early interventions benefit the mental health, academic performance and productivity of children and adolescents throughout their life. The present study protocol will evaluate the effectiveness of the Elos 2.0 Programme, which is a version adapted for Brazil by the Ministry of Health, in reducing problem behaviours (e.g., disruptiveness, aggressivity and shyness) and promoting social skills in the school context in children 6 to 10 years of age. The Elos Programme is based on the Good Behaviour Game*,* which is widely used and prevents and/or reduces students’ disruptive behaviours by establishing cooperation contingencies.

**Method:**

A cluster-randomized controlled trial will be performed in 30 schools in three cities (15 controls and 15 in the experimental group), with a total of 3800 children participating in the test (1900 in the control group and 1900 in the intervention group). Data will be collected by having teachers in the control and experimental classes complete the Teacher Observation of Classroom Adaptation (TOCA) questionnaire, which is an instrument used to observe children’s behaviour in the classroom. We will collect data before and after the intervention period in the same year. Due to the hierarchical structure of the data, multilevel analysis will be performed to detect simultaneous differences in prevalence over time and across groups to control for sociodemographic variables.

**Discussion:**

The current study will examine the effectiveness of the Elos 2.0 Programme in reducing problem behaviours (e.g., disruptiveness, aggressivity and shyness) and promoting social skills in the school context. The findings of this school-based prevention programme for children will influence the development and implementation of similar programmes for schools and educational policymakers by identifying mechanisms that are central to achieving positive outcomes for participants.

**Trial registration:**

Registry of Clinical Trials of the Ministry of Health RBR-86c6jp. Registered February 2, 2019

**Supplementary Information:**

The online version contains supplementary material available at 10.1186/s13063-021-05408-0.

## Administrative information

The order of the items was modified to group similar items (see http://www.equator-network.org/reporting-guidelines/spirit-2013-statement-defining-standard-protocol-items-for-clinical-trials/).

**Table:** 

Title {1}	Effectiveness of the Elos 2.0 prevention programme for the reduction of problem behaviours and promotion of social skills in schoolchildren: study protocol for a cluster-randomized controlled trial
Trial registration {2a and 2b}.	RBR-86c6jp (http://www.ensaiosclinicos.gov.br/rg/RBR-86c6jp/). Registered 11 February 2019.
Protocol version {3}	February 11, 2019 Original version
Funding {4}	This study received a Research and Innovation Grant for the Prevention of Mental Disorders and Use of Alcohol and other Drugs (“Pesquisas e Inovações em Prevenção de Transtornos Mentais e Uso de Álcool e Outras Drogas”) funded by the Brazilian Ministry of Health (TED #176/2017). The funder’s roles consist of selecting the most suitable grant applications and implementing the Elos 2.0 Programme
Author details {5a}	^1^Department of Psychiatry, Universidade Federal de São Paulo, São Paulo, Brazil; ^2^School of Public Health, The University of Hong Kong, Hong Kong SAR, China, ^3^Department of Clinical Psychology, University of São Paulo, São Paulo, Brazil; ^4^Department of Preventive Medicine, Universidade Federal de São Paulo, São Paulo, Brazil
Name and contact information for the trial sponsor {5b}	Brazilian Ministry of Health Address: Esplanada dos Ministérios, Bloco G, Edifício Sede, Brasília - DF, Brazil, 70058-900 https://www.gov.br/saude/pt-br +55 (61) 3315-3580/2005
Role of sponsor {5c}	The funding agency is not involved in the design of this study and will have a role during its implementation, but not in the analyses, interpretation of the data and decision to submit results. The sponsor declares that it has no commercial interest or sources in the application of this trial.

## Introduction

### Background and rationale {6a}

One fifth of children worldwide are affected by emotional and behavioural problems. The disease burden associated with these mental disorders includes the costs of their reduced chance of completing basic education, lack of social cohesion and reduced ability to cope with future adversities, which are also associated with negative outcomes later in life if no preventive measures are taken [1–4].

Interventions in childhood generally aim to promote the overall development of children. However, evidence shows that these early interventions also benefit the short- and long-term mental health of children and adolescents [2, 5]. Studies examining the effects of preventive interventions in educational or family contexts on children and adolescents with some risk factors showed positive results in reducing the incidence and/or progression of mental disorders throughout life [6–9].

The Good Behaviour Game (GBG) is one of the most effective programmes to promote mental health in children and adolescents and prevent and/or reduce students’ disruptive behaviours via the establishment of cooperation contingencies [10, 11]. The GBG aims to equip teachers with behaviour management strategies in the classroom to reduce aggressive or disruptive behaviours, shyness and/or social isolation. These behaviours are risk factors for future negative outcomes in mental health, such as anxiety and depression [12].

The GBG procedure is divided into three stages. In the first stage, students from the same classroom are divided into heterogeneous teams according to gender, academic performance and behaviour pattern (problems and prosocial). The teachers presents four rules that must be followed during GBG: (1) follow the instructions for activities, (2) follow the voice levels (silence, whisper, group voice, presentation or street voice), (3) follow the arrangement of seats (remain seated, stand and walk as agreed or stand and walk freely) and (4) be kind. The rules are illustrated on posters posted in classrooms as the game takes place. To play GBG, teachers must select pedagogical activities according to the school curriculum that students can perform autonomously, such as exercises and artistic activities. Therefore, the teacher does not offer pedagogical support to the students during the GBG to promote autonomy and group cooperation.

The teacher observes the students during the GBG, and if they break the rules, he/she (1) describes the inappropriate behaviour to the classroom in a neutral tone of voice immediately after the occurrence, (2) records the rule break on the blackboard and assigns one point and (3) praises the other students who are following the rules.

At the end of GBG, teams with a maximum of five points win the game. Notably, all of the teams can win the game, which avoids the existence of competition between children. The teachers announce the winning teams but do not mention the losing teams. The winning teams and students receive prizes, including tangible immediate reinforcers, such as stamps in a notebook and coloured pencils, and intangible immediate reinforcers, such as praise and applause. There is also a score for teams that win games over a period of time, such as a week, for the distribution of delayed reinforcers. Briefly, the awards and recognitions for following the rules occur in three ways: reinforcement to the students immediately after the game, reinforcement to the team immediately after the game and reinforcement to the team at the end of each week of the game [13–15].

Since its inception, the GBG has been implemented in several countries, including the USA [13], Sudan [16], the UK [17] and Belgium [18, 19]. Because the GBG has been widely used, it is an evidence-based practice with proven effectiveness for the (a) reduction of disruptive and aggressive behaviours in the school context [11], (b) prevention of the use and abuse of psychoactive substances [12, 20, 21], (c) prevention of suicide ideation and attempts [20, 22] and (d) prevention of the development of violent behaviour and antisocial personality disorder in adulthood [23].

The integration of GBG in the school curriculum is an innovative proposal that could facilitate the teaching and learning processes [24]. Because it improves interactions between teachers and students, the GBG likely improves students’ mental health outcomes and enhances their academic abilities. Positive teacher-student social interactions are associated with better academic results because the teacher builds a welcoming environment via the use of social reinforcers, such as acknowledgements, praise and descriptions of appropriate behaviours [25–27]. Good academic results in children and adolescents, especially results related to reading, contribute to good mental health indicators, such as empathy [28], rationality and creativity [29], social well-being, well-defined daily routine and self-satisfaction with life [30, 31].

Previous studies demonstrated that teacher-specific characteristics, such as years of experience, education, mental health and a sense of self-efficacy, tended to impact social interactions and school climate [25, 32, 33]. These characteristics are relevant because the teacher profile may attenuate problem behaviours in students by promoting academic success and social skills, which would produce a compensatory effect on the maladaptive behaviour losses [34–36].

The GBG programme was adapted in Brazil in 2013 for children between 6 and 10 years of age and named “Programa Elos: construindo coletivos” (Elos Programme: constructing collectives). The objective of the programme is to promote cooperative and democratic interactions between teachers and students [37]. By changing interactions that are considered maladaptive in the classroom context, the programme intends to promote mental health and prevent and/or reduce problem behaviours (e.g., disruptiveness, aggressivity and shyness). The purpose of the Elos Programme is also to promote early protective factors to eliminate or minimize the risks of drug use and abuse in the long term. In other words, it is a tool for the prevention of the use and abuse of psychoactive substances, which was shown in other studies of GBG [12, 23].

The cultural adaptation and implementation of the Elos Programme pre-pilot occurred in four Brazilian municipalities in 2013. The programme was implemented in other schools in 17 municipalities on a pilot basis in subsequent years (2014 and 2015) [38]. However, randomized controlled trials with a control group to test the effectiveness of this programme in Brazil were not performed. Therefore, the programme materials were revised, and the updated version of the programme was named Elos 2.0.

To disseminate this tool on a national scale, it is essential to test the effectiveness of the programme when it is adapted to our culture, as recommended by the PROMISE (Providing mental health promotion training guidelines and training resources for healthcare professionals) project [39]*.*

## Objectives {7}

The main objective of this cluster-randomized controlled trial is to evaluate the effectiveness of the Elos 2.0 Programme, which is a version adapted to Brazil for proposed implementation by the Ministry of Health, in the reduction of problem behaviours and promotion of prosocial behaviours in children in public schools. All outcomes are measured at the level of the individual child. Our main hypothesis is that students between the first and fourth years of elementary school who are exposed to the Elos 2.0 programme for 5 months will have a lower frequency of problem behaviours (e.g., disruptiveness, aggressivity and shyness) and better prosocial behaviours compared to the control group.

## Trial design {8}

A cluster-randomized controlled superiority trial will be performed in 30 schools in three cities (15 controls and 15 in the experimental group), with a total of 3800 children participating in the test (1900 in the control group and 1900 in the intervention group). This study is a randomized controlled trial with two parallel groups (intervention and control) of students aged 6 to 10 years and enrolled in the first to fourth year of elementary education in public schools in the cities of Fortaleza, Eusébio and São Paulo, which are located in the states of Ceará and São Paulo. The ratio of intended numbers of participants in each of the comparison groups is 1:1. The control group receives treatment as usual in Brazil, i.e., no behavioural intervention at the school.

## Methods: participants, interventions and outcomes

### Study setting {9}

Public schools with at least one class of each grade (first to fourth grade) will be included. Schools enrolled in the trial will not offer programmes and/or activities for the prevention of drug use and abuse or the promotion of mental health in 2019. Collaboration with the Municipal and State Departments of Health and Education, which is essential for the implementation and evaluation of the Elos 2.0 Programme in schools, will be led by the National Coordination of Mental Health, Alcohol and Other Drugs of the Ministry of Health (responsible for implementing the programme).

### Eligibility criteria {10}

Schools will be included in the study according to the following criteria: (1) being a public school and (2) having at least one class of each grade (first to fourth grade) and all children in these grades will be included in the trial.

### Who will take informed consent? {26a}

Study coordinators and trained interviewers will introduce the trial to participants, who will attend a seminar on the main aspects of the trial. Participants will also receive information sheets. Participants may have an informed discussion with the study coordinator and staff. Trained interviewers will obtain written consent from participants who are willing to participate in the trial. Information sheets and consent forms will be provided for all participants involved in the trial, but these forms were amended accordingly to provide separate information sheets and consent forms, which are more suitable for children. We will obtain consent to participate in the study from the schools’ principals, teachers, students and parents. The intervention will be part of the school curriculum, and it will be mandatory for all students in the active school group.

### Additional consent provisions for the collection and use of participant data and biological specimens {26b}

Not applicable. This trial does not include the collection or derivation of data for purposes that are separate from the main objective of study.

## Interventions

### Explanation for the choice of comparators {6b}

Among the schools selected to participate in the study, a second simple randomized selection will define the control and experimental schools at a ratio of 1:1 control and experimental schools per municipality. The selected comparison group is treatment as usual, which means no behavioural interventions delivered in the control schools. This is a suitable comparator, as it is the standard experience of schoolchildren in Brazil. Public schools for children in Brazil do not usually receive interventions to improve behavioural skills.

### Intervention description {11a}

Teachers will present four rules to be followed by the student teams during the Elos Game 2.0: (1) follow the instructions for the activities, (2) adhere to the voice levels (silence, whispering, group voice, presentation or street voice), (3) comply with assigned positions (remain seated, stand up and walk as arranged or stand up and walk freely) and (4) be kind. The rules will be the same for all teachers and will be illustrated on posters that will be posted in classrooms. To play the Elos Game, teachers should select pedagogical activities that are consistent with the school curriculum and can be performed autonomously.

For each experimental school, all students from the first to fourth grades will participate in the Elos Programme as a regular school activity, and the school will designate one teacher per class to receive training on the programme.

### Criteria for discontinuing or modifying allocated interventions {11b}

The assigned study intervention will be offered equally to all classes enrolled in the intervention group. If any teacher or student refuses the invitation to participate in the trial or refuses to discuss the process of implementation, they will not be included in the study, since agreement to participate is an inclusion criterion. If teacher refuses the invitation to participate in the trial, we will reallocate other school for the intervention group following the random drawing from the list. Teachers and students who started the trial but decide to drop the study after baseline assessment they will provide follow-up data.

### Strategies to improve adherence to interventions {11c}

We will construct social media groups with teachers to keep in touch.

### Relevant concomitant care permitted or prohibited during the trial {11d}

We will explain the concomitant care and interventions that are prohibited during the trial to the teachers.

### Ancillary and post-trial care {30}

We will provide ancillary and post-trial care at local child mental health outpatient units at the Universidade Federal do Ceará, in Ceará, and UNIFESP in São Paulo.

### Outcomes {12}

Main outcome is as follows: child problems and prosocial behaviours. All outcomes will be measured at the individual level, but analyses will be multilevel based on schools and individuals. The main outcome is a variable compound of three measures (child concentration, disruptive problem and prosocial behaviour) that are measured using only one instrument, the Teacher Observation of Classroom Adaptation-Checklist (TOCA-C). The main outcome measure is completed twice for each child by teachers with mediation of a trained interviewer: at baseline (before trial is implemented) and after trial completion.

### Participant timeline {13}

**Table:** 

SPIRIT schedule							
Forms and procedures				Study period			
Activity	CRF (Y/N)^a^	Staff	Time to complete (minutes)	− T1	0	T1	T2
Eligibility screen	N	Study coordinator	5	X			
Informed consent	N	Interviewer	5	X			
Baseline randomization	N	Study statistician	5	X			
Allocation	N	Study coordinator	5	X	X		
Teacher training	N	All members	360		X		
**Intervention**							
Elos 2.0 Programme	N	Trained teachers	480–2400			X	
Teacher supervision	N	Trained supervisor	120			X	
Supervisor supervision	N	Study coordinator	240			X	
**Assessments**							
Teacher training	N	Interviewer	10		X		X
Bullying	N	Interviewer	15		X		X
Field checklist	N	Trained supervisor	5			X	
Fidelity	N	Trained supervisor	5			X	
Language and math	N	Interviewer	20		X		X
School observation	N	Interviewer	20		X		X
Teachers protocol	N	Interviewer	15		X		X
Game register	N	Trained supervisor	5			X	
SDQ	N	Trained teacher with mediation of a trained interviewer	15		X		X
STRS	N	Trained teacher with mediation of a trained interviewer	15		X		X
TOCA-C	N	Trained teacher with mediation of a trained interviewer	15		X		X

^a^Case report form

### Sample size {14}

Our target between group mean difference for TOCA scores was 0.25, which we believe to be a plausible treatment effect for this intervention, and would be clinically meaningful. A sample size of *N* = 3000 child participants (1500 in each treatment group), comprising 15 clusters with an average of 100 children per cluster, is sufficient to provide 92% power, with an alpha threshold of 0.05, a standard deviation of 0.80 based on the study by Storr et al. [40], an intraclass correlation coefficient of 0.05 (a conservative value compared with a previously reported value of 0.039 by Liljequist et al. [41]), and setting the coefficient of variation of cluster sizes at 0.70.

### Recruitment {15}

In collaboration with the Municipal and State Departments of Health and Education, we will select schools for inclusion in the study according to the following criteria: (1) being a public school and (2) having at least one class of each grade (first to fourth grade). Each city will have its own list of schools. We will randomly select schools for a specific city from each list, and the eligible schools will be invited to participate in the trial. However, the schools may refuse to participate in the trial. In case of refusal, we will randomly select 10% more schools to replace the schools that refused to participate.

## Assignment of interventions: allocation

### Sequence generation {16a}

The allocation of schools (1:1) to the control or intervention group will be performed via simple random drawing from the list of the Anísio Teixeira National Institute of Educational Studies and Research (INEP, its acronym in Portuguese), which includes all Brazilian schools separated by city. Randomization will be performed using a quantitative methodology in PASS 15 Efron’s biased coin (two treatments, equal sample sizes). The target sample sizes are the same for both groups. To achieve a longitudinal balance between groups, the algorithm dynamically changes the group assignment probabilities.

The unit of randomization between groups is the schools and not the classes. Therefore, there will be no control classes in an experimental school and vice versa to avoid sample contamination. Agreement to participate in the study was received from the schools before randomization.

### Concealment mechanism {16b}

Only 30 of the eligible schools will join the trial based on our pre-estimated sample size. The initial selection of 30 schools will use www.random.org. All of the schools’ names will receive a number from one to the total number of eligible schools (i.e., if there are 42 eligible school names, then each one will receive a number from 1 to 42). A random number generator will select 30 numbers from a range of 1 to the total number of eligible schools. These 30 numbers will be randomly assigned to control or intervention groups using Efron’s biased coin to ensure balanced groups. All schools recruited in the same city will be randomized simultaneously. Randomization within each city will be performed when all schools are recruited for that city, rather than waiting until all schools (across all cities) are recruited and then performing randomization of all schools simultaneously. Members of the trial team who are involved with recruitment of schools will be unaware of previous allocations. Trial researchers, not involved with school recruitment, will inform schools of their randomized allocation.

### Implementation {16c}

All schools from the 30-school lists that give consent for participation and fulfil the inclusion criteria will be randomized. The staff member responsible for the recruitment and baseline interviews will request the randomization. The Ministry of Health team will implement all stages of the intervention, including the recruitment, informing of intervention treatment and supervision.

## Assignment of interventions: blinding

### Who will be blinded {17a}

Due to the nature of the intervention, neither teacher participants nor school staff can be blinded to the allocation. Data collection team will be blind to which group (control or intervention) the school will be allocated. We will not directly provide information on the school allocation to the external assessors who will interview the teachers, but no one is truly blinded in this type of randomized controlled trial because the teachers may accidentally report the school status.

### Procedure for unblinding if needed {17b}

Not applicable. This trial does not have the potential to harm or other relevant conditions that necessitate an unblinding protocol prevention.

## Data collection and management

### Plans for assessment and collection of outcomes {18a}

The main outcome of the study is the behavioural repertoire of the children, including problem behaviours, such as disruptiveness, aggressiveness and shyness, and prosocial behaviours. We will control for covariates, such as the gender and age of teachers and children, and the teachers’ stress levels and senses of self-efficacy. The main outcome will be collected using the TOCA-C, which is an instrument to measure the social adaptation behaviours of elementary school students. The teacher answers the 21 items of the instrument and classifies the frequency of each student’s behaviours in the classroom on a Likert scale (ranging from “never” to “almost always”). The TOCA-C was designed to investigate disruptive behaviours or problems with concentration and social skills [42]. The instrument was translated by the team of researchers responsible for the project, who will also perform validation and cultural adaptation to Brazil. Covariates will be collected using the Student-Teacher Relationship Scale-Short Form (STRS-SF), which is a sociodemographic questionnaire on age, gender, education and length of professional experience. Teacher socioeconomic status will be collected using the Index of the Brazilian Association of Research Companies. Sense of competence will be assessed using the Bäbler and Schwarzer General Self-Efficacy Scale as adapted by Gil-Monte and Moreno-Jiménez. Teachers’ mental health will be assessed using the Self-reporting Questionnaire (SRQ 20).

### Plans to promote participant retention and complete follow-up {18b}

A study supervisor and trained supervisor will provide periodic communication via weekly meetings and presentations to inform the school officials/staff, students and parents about the Elos 2.0 Programme, the current status of the programme and plans for the next phase. These supervisors will also acknowledge their support in periodic meetings and motivational posts on social media.

### Data management {19}

In the Elos 2.0 Programme, all data from the Elos 2.0 Programme will be entered in two stages: (1) interviewers will enter documents in the proper form and (2) all of the data will be entered electronically. These entries may be done at the participating site where the data was originated. Original study forms will be entered and kept on file at the participating site. A subset will be requested later for quality control. When a form is selected for review, the participating site staff will pull that form, copy it and send the copy to the study supervisor for re-entry. Participant files will be stored in numerical order in a secure and accessible place and manner. All forms related to study data will be kept in locked cabinets and access to the study data will be restricted to the study supervisor and assigned data management member staff. Participant files will be maintained in storage for three years after completion of the study. Errors will be detected by the study supervisor and data management member staff who are assigned to detect missing data or specific errors in the data.

### Confidentiality {27}

All study-related information will be stored securely at the study site. All participant information will be stored in locked file cabinets in areas with limited access. All data collection, processes and administrative forms will be identified using a coded ID [identification] number only to maintain participant confidentiality. All records that contain names or other personal identifiers, such as locator forms and informed consent forms, will be stored separately from the study records identified by the code number. Forms, lists, logbooks, appointment books and any other listings that link participant ID numbers to other identifying information will be stored in a separate, locked file in an area with limited access.

### Plans for the collection, laboratory evaluation and storage of biological specimens for genetic or molecular analysis in this trial/future use {33}

Not applicable. No biological specimens will be collected as part of this trial.

## Statistical methods

### Statistical methods for main outcomes {20a}

Four two-level regression analyses for the observed continuous dependent measures will be performed (one analysis for each subscale of the TOCA and one for TOCA general score; disruptiveness, concentration problem and prosocial behaviour). Each grade has a teacher who is responsible for the implementation of the Elos 2.0, and consequently, teacher and classroom effects are not dissociable. There is no cross-classification between teachers and classrooms (i.e., a teacher for the first grade will be solely responsible to this group of students). Moreover, all the classroom within the same school receives the same intervention (i.e., Elos 2.0 or treatment as usual).

This multilevel analysis will be dealt on Mplus version 8.4 under the maximum likelihood with robust standard errors (MLR). This estimator allows dealing with the non-independence of the observation (i.e., children nested classrooms); the standard error will be computed considering such multilevel structure by command in Mplus called (TYPE = Complex) using a sandwich estimator where the cluster variable will the classroom (i.e., teachers level) [43]. Adopted statistical significance was 0.05. Important to note that although within the school with have different classroom, all the classrooms within the same school are receiving either Elos 2.0 or treatment as usual to avoid contamination among the teachers. Therefore, our analysis will be following a two-level approach. In case of moderate or strong differences or unbalanced proportions between the control/intervention schools and/or children features on baseline measurements, the later will be inserted as covariates in the model for estimating the intervention effects. The only exception will be the baseline values of the outcome variables, which will be included in the within part as a predictor of the targeted outcome. This approach is also called the residualized change score [44].

### Interim analyses {21b}

Not applicable. This trial does not have a longer duration of recruitment or potentially serious outcomes. However, the Brazilian Ministry of Health can audit our protocol anytime.

### Methods for additional analyses (e.g., subgroup analyses) {20b}

For qualitative data, we will perform content analyses based on grounded theory using the NVivo 10 software programme. For qualitative variables, the summary measures with be the number and percentage. For numerical variables, the summary measures will be the mean, standard deviation, median, minimum and maximum. The integration of parallel databases will be performed via pairing of the secret codes using the Levenshtein algorithm. There is no analysis planned for any subgroup to identify interactions between the intervention and baseline participant characteristics, such as age or gender.

### Methods in the analysis to handle protocol non-adherence and any statistical methods to handle missing data {20c}

We will compose a letter-number code for each subject based on the school and personal information to maintain confidentiality and blinding in the data analyses. We will perform double data entry in the dataset and multiple imputation to handle missing data from the follow-up of participants which do not refuse to participate at the beginning or do not decline to perform the implementation. Missing data in the follow-up could occur if the child misses school on the data collection or if the child changes of school or city, which is likely to occur in any longitudinal drawing. Two different techniques will be used and contrasted: multiple imputation and the complier-average causal effect (CACE), which provide a form of sensitivity analysis of our intervention effects [45, 46]. CACE will be calculated by using data from the fidelity forms answered weekly by the teachers. Non-adherence will not consider the classroom where the teacher did not complete the program or have not delivery at least 50% sessions of Elos.

Multiple imputation operates under the assumption that the underlying missing mechanism is *missing at random* (MAR) and here multiple imputation will be carried out using Bayes estimation of an unrestricted variance-covariance model, where all of the main assessments of baseline sociodemographic and school features in the data set will be assumed as dependent variables. The variables with missing values will be included in the imputation step. The number of data sets to be imputed will depend on the fraction of missing information [42]. In case of missing data on child primary outcome (follow-up), they will be imputed. We do not expect child missing data at baseline outcome, but situation as loss of questionnaires, parents giving up of the children participations might happen requiring imputation of baseline primary outcome assessment. Multiple imputation is in accordance with intention-to-treat paradigm (ITT) broadly advocated by CONSORT [44]. We will perform an analysis using the ITT principle (all participants will be analysed as randomized, irrespective of whether they received the intervention in the intervention group) with observed data only, and a further ITT analysis using observed and imputed data. The primary analysis will be with the ITT using observed data only.

To deal with missing data from teachers who decline to participate after the training and partial implementation (i.e., absence of answers in questionnaires) CACE will be contrasted to the traditional intention-to-treat approach [47]. CACE involves a mixture modelling [48], which allows us to robustly estimate the effect of the intervention among those who were compliers in the intervention group versus those who were potential compliers among those in treatment as usual group. The status of adherent (or not) is not directly observable among those in control group because the participants were not exposed to the intervention. Therefore, one should use latent variable modelling to uncover the status of the participants in such a group. Major details about CACE modelling might be found in [49, 50]. As a sensitivity analysis, a CACE analysis will be performed using the adherence data for each teacher (i.e., each teacher and pupil in the intervention group will be considered as a “complier” or “non-complier”).

### Plans to allow access to the full protocol, participant-level data and statistical code {31c}

No later than 3 years after the collection of the 1-year follow-up interviews, we will deliver a completely deidentified data set to an appropriate data archive for sharing purposes.

## Oversight and monitoring

### Composition of the coordinating centre and trial steering committee {5d}

The study design and grant protocol were drafted by SCC and ZMS. MM and ARS were responsible for drafting the article. JLSL and NTP were responsible for the literature review. HCM performed the sample size calculation and the school randomization. HCM, MHSM and JJM were critical reviewers of the study design and the present manuscript. There is no fixed external trial steering committee, but the Brazilian Ministry of Health may audit our protocol and have experts in any field (e.g., education, psychology, trial methodology and statistics) review our protocol.

### Composition of the data monitoring committee, its role and reporting structure {21a}

The protocol will not have a fixed data monitoring committee because this trial has a short duration and known minimal risks. However, the Brazilian Ministry of Health may perform an audit and have experts in any field (e.g., education, psychology, trial methodology and statistics) review our protocol at any time.

### Adverse event reporting and harms {22}

There are no reports of adverse effects of the intervention in the literature. The supervision that teachers will provide throughout the intervention is also focused on discussing the possible negative effects of the game for children to prevent adverse effects. According to the Brazilian Research Ethics Committee, for people who accept to participate and sign the consent terms are guaranteed treatment for adverse effects in the next 5 years. If the participants report adverse effects of unintended consequences of the intervention, they will be assisted in the treatment by the Ministry of Health’s intervention implementation team.

### Frequency and plans for auditing trial conduct {23}

The Brazilian Ministry of Health may audit the study at any time.

### Plans for communicating important protocol amendments to relevant parties (e.g., trial participants and ethical committees) {25}

Any amendments, including administrative amendments, will be approved by the Ethics Committee of the State Health Secretary (CAAE 01517218.2.0000.5505, CEP/UNIFESP no. 1246/2018) prior to implementation, and the health authorities will be notified in accordance with local regulations.

## Dissemination plans {31a}

All presentations and publications will protect the integrity of the major objectives of the study. Data that break the blinding will not be presented prior to the release of the main results. The study results will be released to the participants and the general community.

### Authorship {31b}

The authors will be responsible as detailed in the following:

- Abstract and main text of articles: MM, ARS and SCC
- Revision of abstract and main text of articles: JLSL, NTP, HCM, MHSM, JJM and ZMS.

### Reproducible research {31b}

This programme is the property of the Brazilian Ministry of Health, which will be responsible for its large-scale dissemination.

## Discussion

This article presents a randomized controlled trial of the Elos 2.0 Programme to evaluate its effectiveness in reducing problem behaviours (e.g., disruptiveness, aggressiveness and shyness) and promoting social skills in the school context. This school-based prevention programme for children between 6 and 10 years of age is based on the GBG. Longitudinal studies showed that the GBG is a “behavioural vaccine” against future risk behaviours [10]. For example, several studies reported that participation in GBG resulted in a lower risk of substance abuse and dependence [12, 51] and decreased frequency of delinquency, juvenile violence and other behavioural disorders among participants [17, 52].

Despite these associations of the problem behaviours in school-age with a wide range of adverse psychosocial outcomes (e.g., crime, substance use and mental health) and the burden that it generates for caregivers and teachers, the mental health of children is neglected, especially in low- and middle-income countries (LMICs) [2]. Prevention programmes, such as the Elos 2.0 Programme, that may be adapted and tested for effectiveness are necessary, particularly in LMICs, where a tremendous gap remains between needs and the available resources [53].

### Strengths and weaknesses

This randomized trial will be performed in a representative sample of public school students in Brazil. We highlight that the randomization will be performed at the school level to ensure that there is no contamination, which can occur when the randomization is performed at the level of the classrooms within the school.

There are several limitations. For example, the intervention will not be repeated if a child is absent, but this fact is a limitation of prevention intervention designs in general [54]. We will be unable to directly evaluate children’s behaviour due to the high cost of these observations. However, the most common method used to evaluate children’s behaviour and functioning at school is the use of rating scales completed by teachers. Teachers’ ratings are predicted by the student’s gender, race/ethnicity, academic performance, disciplinary incidents, the teacher’s gender, student-teacher gender interaction, teacher professional development in behaviour screening and classroom academic performance [55].

### Trial status

 Protocol version = 1

Date of register = February 2019

Date recruitment began = March 2019

Estimate completion date = November 2020

## Supplementary Information

**Additional file 1.** Informed Consent Form for Teachers. Informed Consent Form for Parents and Guardians. Term of Consent for Students. Informed Consent Form for School Principals. Consent to participate in the studies.

## Abbreviations

GBG: Good Behavioural Game
INEP: National Institute for Educational Studies and Research “Anísio Teixeira”
REBEC: Registry of Clinical Trials of the Ministry of Health
