# Supplementary material for: Effectiveness of the Elos 2.0 prevention programme for the reduction of problem behaviours and promotion of social skills in schoolchildren: study protocol for a cluster-randomized controlled trial
Source: Trials. 2021 Jul 20;22:468. doi: 10.1186/s13063-021-05408-0 (PMC8290592; doi:10.1186/s13063-021-05408-0)
Supplement: Supplementary file 1 — Additional file 1. Informed Consent Form for Teachers. Informed Consent Form for Parents and Guardians. Term of Consent for Students. Informed Consent Form for School Principals. Consent to participate in the studies. [file 13063_2021_5408_MOESM1_ESM.docx]

**Informed Consent Form for Teachers - 1/2**

Name of teacher _____________________________________________________

Name of school: _________________________________________________________

**Study: Evaluation of the effectiveness of the Elos Programme - Building Collective to reduce aggression in children from the first to the fourth grade of elementary school.**

Your student and yourself are invited to participate in a research project. Before you decide to participate in this study, please read this document carefully and ask any questions you may have to make sure that you understand how the study will be performed.

This study aims to assess the effectiveness of the Brazilian version of the *Elos* school programme in the implementation model proposed by the Ministry of Health in the prevention of aggressive behaviour and excessive shyness in students during the first to fourth years of public elementary school.

Participation in the study involves the evaluation of documents recording activities in the classroom and participation in the completion of questionnaires on a school prevention programme implemented by the Ministry of Health and the Ministry of Education within the scope of the School Health Programme PSE (Programa Saúde na Escola). Please note that the completion of these documents is anonymous, and the information provided will be used exclusively for research purposes. The information obtained will be analysed and published in a printed report for release by the Ministry of Health and Ministry of Education at the end of the study.

Your participation is voluntary and may be withdrawn at any time. It should be clarified that participation does not involve any direct benefit to students or teachers, and there are no expenses or financial compensation for participation.

However, participation may benefit the entire community because the results of these studies aim to support effective drug use prevention programmes in Brazilian schools in the long term.

Notably, the study ensures the anonymity of the participating schools and all students and teachers interviewed. Under no circumstances will the schools, students or teachers participating in the study be identified.

At every stage of the study, you will have access to the professionals responsible for the research to clarify any doubts. The principal investigator is Professor Dr. Sheila C. Caetano, and you may contact her by telephone (11) 3466-2170, e-mail: sheila.caetano@unifesp.br, or at the address: Major Maragliano, 241, Vila Mariana - São Paulo - SP.

If you have any questions or concerns about the research, please contact the Research Ethics Committee of UNIFESP (Rua Prof. Francisco de Castro, n: 55, -CEP: 04020-050 – tel 5571 1062- fax 5539 7162 – e-mail: cepunifesp@epm.br). Telephone and in-person hours are available on Mondays, Tuesdays, Thursdays and Fridays from 9 am to 1 pm.

This document is being made available in two original copies: one copy must be delivered to the participant, and the other copy will remain with the responsible researcher (Resolution CNS No. 466 of 2012, item IV.5.d).

**Informed Consent Form for Teachers - 2/2**

**Consent to participate in the studies**

*I, _____________________________________________________ believe that I was sufficiently informed about the study “* ***Evaluation of the effectiveness of the Elos Programme - Building Collective to reduce aggression in children from the first to the fourth year of elementary school”*** *and discussed with the researcher my decision to authorize participation of this school in these studies.*

*The purposes of the studies, the procedures to be performed, the guarantees of confidentiality and permanent clarifications are clear. I understand that participation is free of charge.*

*I voluntarily consent to my participation with knowledge that I can withdraw my consent at any time, before or during the study, without penalties or losses.*

__________________________________________________________

**Name of participant**

*I declare that I have appropriately and voluntarily obtained the consent of the person responsible for the participation of this institution in these studies.*

__________________________________________________________

Professor Dr. Sheila Cavalcante Caetano

**Name and signature of the researcher**

**Informed Consent Form for Parents and Guardians - 1/3**

Dear parent or guardian,

Your child’s school is participating in the study *“Evaluation of the effectiveness of the programme for the reduction of aggression in children from the first to the fourth grade of elementary school of the Ministry of Health: Elos 2.0 Programme, version 2019”, after* the principal agreed to participate. However, we need your consent and approval to use the responses of your child. Therefore, we are sending this document entitled “Informed Consent Form”. Before you decide to authorize your child’s participation in this study, please read this document carefully.

This study is a randomized controlled trial to evaluate the outcome of a programme that aims to reduce aggression in children from the first to the fourth grades of elementary school, called the Elos 2.0 Programme. The study has two groups: one group will receive the programme, called the experimental group, and the other group will not receive the programme, called the control group. Your child may participate either group according to a randomized school draw. Students in both groups will receive an ANONYMOUS multiple-choice questionnaire that will take approximately 45 minutes to complete. The questionnaire will be used twice in 2019: once in March 2019 (pre-test) and once in November 2019 (post-test).

Children it the experimental group will participate in games performed in the classroom led by the teacher and supervised by the Ministry of Health staff during class hours. These games will include activities that are already planned by the pedagogical planning of the school, and no extra activities will be added. During the game, the students will follow rules that involve the level of voice used, being kind to other colleagues and following instructions on how to perform the proposed activity.

The questions in the questionnaire will be about reading and vocabulary skills, social skills and bullying. The questionnaire is anonymous, that is, no one will ever know who answered it. Everything that your child completes in the questionnaires will be confidential and will be used only for the purpose of this study. In other words, your child’s name will not be attached to any questionnaire, report or presentation about this study.

The objective of this study is to allow the Ministry of Health, as the developer of the programme, to assess whether it reduces aggressiveness and inadequate

**Informed Consent Form for Parents and Guardians - 2/3**

behaviours, helps in the acquisition of prosocial skills and increases task engagement. These results may act as protective and preventive factors against the non-use of alcohol and drugs when the children are older. Therefore, the main justification is the need for studies to define the public policies of our country. It is necessary to know whether the programmes truly work and if they are being well implemented in schools.

Participation is voluntary and may be withdrawn by you or your child at any time. Participation does not provide a direct benefit to the participants, and there are no expenses or financial compensation for participation. However, it may benefit the entire community where he/she studies and lives because we plan to use the results of this study to support good drug use prevention programmes in Brazilian schools.

You are also invited to participate in the study. Your participation consists of completing an ANONYMOUS multiple-choice questionnaire that will take approximately 45 minutes to complete. The questions in the questionnaire are about you, your child, your family and how they feel and behave.

Participation in this study will not harm anyone in any way. In addition, you have the choice whether or not to complete the questionnaire, and if you and your child do not want to complete it, you will not be punished or reprimanded. You can decide not to participate or decide not to finish the questionnaire even after you begin.

If your child needs any psychiatric assistance, you can contact the mental health support clinic DICA (Integral Development of Children and Adolescents of the Department of Psychiatry of the Paulista School of Medicine of UNIFESP) located at Rua Major Maragliano, 241, Vila Mariana (São Paulo) by telephone (11) 3466-2170. This outpatient clinic is coordinated by the professor at UNIFESP, Dr. Sheila C. Caetano.

**People to Contact**

At every stage of the study, you will have access to the professionals responsible for the study to clarify any doubts about the research procedures. The principal investigator is Professor Sheila C. Caetano, who can be found at the Psychiatry Department of UNIFESP (Rua Major Maragliano 241, - tel: (11) 3466-2170. - e-mail: sheilaccaetano@gmail.com). If you have any questions or concerns about the study or want to report ethical irregularities, contact the Research Ethics Committee of UNIFESP (Rua Francisco de Castro, 55, tel 11-5571 1062 - e-mail: cepunifesp@epm.br) and the Research Ethics Committee of the Municipal Health Secretariat (Rua General Jardim, 36, 8th floor, Vila Buarque, São Paulo/SP. CEP 01018-001. Phone: (11) 33972464, e-mail: smscep@gmail.com).

**Informed Consent Form for Parents and Guardians - 3/3**

**Consent to Participate**

*I, _____________________________________________________, responsible for the student __________________________________________ after having been duly informed about the study “Evaluation of the effectiveness of the programme for the reduction of aggression in children from the first to the fourth grade of elementary school Ministry of Health: Elos 2.0 Programme, version 2019 ”,* ***accept that he/she participates in the study.***

_______________________________

Name and signature of the participant

Date:

I, Sheila Cavalcante Caetano, *declare that I have obtained the appropriate and voluntary consent of the person responsible for participation in this study.*

_________________________________

Professor Dr. Sheila Cavalcante Caetano

**Term of Consent for Students - 1/3**

Dear student,

You are being invited to participate in a study called *“Evaluation of the effectiveness of the programme for the reduction of aggression in children from the first to the fourth year of elementary school of the Ministry of Health: Elos 2.0 Programme, version 2019”*, which is a programme to reduce aggression and inappropriate behaviours, promote the acquisition of prosocial skills and increase task engagement that is being implemented in some public schools.

If you decide to be a part of the study, we will ask you to complete a multiple-choice questionnaire that will take approximately 45 minutes to complete. The questionnaire will be given to you twice: once in March 2019 (pre-test) and once in November 2019 (post-test).

The questions on the questionnaire are about your school environment, reading skills, vocabulary and some behaviours. The questionnaire is anonymous, which means that no one will know that you answered it. Therefore, we ask that you do not put your name anywhere in the questionnaire. In addition, when we tell other people about the results of this study, we will never use your name or the name of your school. Therefore, other people will never know about your participation in the study.

The results of this study may help Brazilian society find a programme in which the skills taught act as protective and preventive factors to reduce the number of children and adolescents who try alcohol and other drugs.

Participating in this study will not harm you in any way. In addition, whether or not you complete the questionnaire is your decision, and no one will be angry with you if you do not want to fill it out. You can stop answering the questions at any time and decide not to finish the questionnaire.

Remember: If you do not want to participate in the study, no one will be angry with you. In addition, if you want to participate now, but change your mind later, you can leave the study at any time. Just leave the questionnaire blank.

If you need any psychiatric assistance, you can contact the mental health support clinic DICA (Integral Development of Children and Adolescents of the Department of Psychiatry of the Paulista School of Medicine of UNIFESP) located at Rua Major Maragliano, 241, Vila Mariana (São Paulo) by telephone

**Term of Assent for Students - 2/3**

(11) 3466-2170. This outpatient clinic is coordinated by Professor Dr. Sheila C. Caetano of UNIFESP.

**People to Contact**

At every stage of the study, you will have access to the professionals responsible for the study to clarify any doubts about the research procedures. The principal investigator is Professor Sheila Cavalcante Caetano, who can be found in the Psychiatry Department of UNIFESP (Rua Major Maragliano 241, - tel: (11) 3466-2170. - e-mail: sheilaccaetano@gmail.com). If you have any questions or concerns about the study or want to report ethical irregularities, contact the Research Ethics Committee of UNIFESP (Rua Francisco de Castro, 55, tel 11-5571 1062 - e-mail: cepunifesp@epm.br) and the Research Ethics Committee of the Municipal Health Secretariat (Rua General Jardim, 36, 8th floor, Vila Buarque, São Paulo/SP. CEP 01018-001. Phone: (11) 33972464, e-mail: smscep@gmail.com).

**Consent to Participate**

*I, _____________________________________________________, a student at the school __________________________________________ after having been duly informed about the study “Evaluation of the effectiveness of the programme for the reduction of aggression in children from the first to the fourth year of elementary education of the Ministry of Health: Elos 2.0 Programme, version 2019 ”,* ***agree to participate in the study.***

_______________________________

Name of participant

Date:

I, Sheila Cavalcante Caetano, *declare that I obtained the consent of this student appropriately and voluntarily for his/her participation in this study.*

_________________________________

Professor Dr. Sheila Cavalcante Caetano

**Informed Consent Form for School Principals - 1/2**

**Project:** “ ***Evaluation of the effectiveness of the Elos Programme - Building Collective to reduce aggression in children from the first to the fourth grade of elementary school”***

This study aims to assess the effectiveness of the Brazilian version of the *Elos* school programme in the implementation model proposed by the Ministry of Health for the prevention of aggressive behaviour and excessive shyness in students in the first to fourth years of public elementary school.

Participation in the study involves the evaluation of documents that record activities in the classroom and the participation of teachers in completing questionnaires on the school prevention programme implemented by the Ministry of Health and the Ministry of Education within the scope of the PSE (Programa Saúde na Escola). Notably, the questionnaires are anonymous, and the information provided will be used exclusively for research purposes. The information obtained will be analysed and published in a printed report for release by the Ministry of Health and Ministry of Education at the end of the study.

Participation is voluntary and may be interrupted by the participant at any time. It should be clarified that participation does not involve a direct benefit to students or teachers, and there are no expenses or financial compensation for participation.

However, it may benefit the entire community because the results of these studies aim to support effective drug use prevention programmes in Brazilian schools in the long term.

Notably, the study ensures the anonymity of the participating schools and all students and teachers interviewed. Under no circumstances will the schools, students or teachers participating in the study be identified.

At every stage of the study, you will have access to the professionals responsible for the research to clarify any doubts. The principal investigator is Professor Dr. Sheila C. Caetano, and you can contact her by phone (11) 3466-2170, via e-mail: sheila.caetano@unifesp.br, or at the address: Major Maragliano, 241, Vila Mariana - São Paulo - SP.

If you have any questions or concerns about the research, please contact the Research Ethics Committee of UNIFESP (Rua Prof. Francisco de Castro, n: 55, -CEP: 04020-050 – tel 5571 1062- fax 5539 7162 – e-mail: cepunifesp@epm.br). Telephone and in-person hours are available on Mondays, Tuesdays, Thursdays and Fridays from 9 am to 1 pm.

**Informed Consent Form for School Principals - 2/2**

This document is available in two original copies: one copy must be delivered to the participant, and the other copy will remain with the researcher in charge (CNS Resolution No. 466 of 2012, item IV.5.d).

**Consent to participate in the studies**

*I, _____________________________________________________ believe that I was sufficiently informed about the study “* ***Evaluation of the effectiveness of the Elos Programme - Building Collective to reduce aggression in children from the first to the fourth year of elementary school”*** *and discussed with the researcher my decision to authorize participation of this school in these studies.*

*The purposes of the studies, the procedures to be performed, the guarantees of confidentiality and permanent clarifications are clear. It was also clear that participation is free of charge.*

*I voluntarily agree to consent to the participation of this school, knowing that I will be able to withdraw my consent at any time, before or during the study, without penalties or losses.*

__________________________________________________________

**Name of the participating school**

_________________________________________________________

**Name and signature of the school principal (or his/her representative)**

*I declare that I have appropriately and voluntarily obtained the consent of the person responsible for the institution’s participation in these studies.*

__________________________________________________________

Professor Dr. Sheila Cavalcante Caetano

**Name and signature of the researcher**
